# Peripheral blood mononuclear cell hyperresponsiveness in patients with premature myocardial infarction without traditional risk factors

**DOI:** 10.1016/j.isci.2023.107183

**Published:** 2023-06-19

**Authors:** Jan-Quinten Mol, Julia van Tuijl, Siroon Bekkering, Charlotte D.C.C. van der Heijden, Sander A.J. Damen, Benjamin C. Cossins, Liesbeth van Emst, Tim M. Nielen, Laura Rodwell, Yang Li, Gheorghe A.M. Pop, Mihai G. Netea, Niels van Royen, Niels P. Riksen, Saloua El Messaoudi

**Affiliations:** 1 Department of Cardiology, Radboud University Medical Center, 6525 GA Nijmegen, the Netherlands; 2 Department of Internal Medicine, Radboud University Medical Center, 6525 GA Nijmegen, the Netherlands; 3 Department of Cardiology, Canisius Wilhelmina Hospital, 6532 SZ Nijmegen, the Netherlands; 4 Section Biostatistics, Department for Health Evidence, Radboud Institute for Health Sciences, Radboud University Medical Center, 6525 GA Nijmegen, the Netherlands; 5 Department of Computational Biology for Individualised Medicine, Centre for Individualised Infection Medicine (CiiM) & TWINCORE, joint ventures between the Helmholtz-Centre for Infection Research (HZI) and the Hannover Medical School (MHH), 30625 Hannover, Germany; 6 Department of Immunology and Metabolism, Life and Medical Sciences Institute, University of Bonn, 53115 Bonn, Germany

**Keywords:** Cardiovascular medicine, Molecular mechanism of gene regulation, Components of the immune system, Transcriptomics

## Abstract

An increasing number of patients develop an atherothrombotic myocardial infarction (MI) in the absence of standard modifiable risk factors (SMuRFs). Monocytes and macrophages regulate the development of atherosclerosis, and monocytes can adopt a long-term hyperinflammatory phenotype by epigenetic reprogramming, which can contribute to atherogenesis (called “trained immunity”). We assessed circulating monocyte phenotype and function and specific histone marks associated with trained immunity in SMuRFless patients with MI and matched healthy controls. Even in the absence of systemic inflammation, monocytes from SMuRFless patients with MI had an increased overall cytokine production capacity, with the strongest difference for LPS-induced interleukin-10 production, which was associated with an enrichment of the permissive histone marker H3K4me3 at the promoter region. Considering the lack of intervenable risk factors in these patients, trained immunity could be a promising target for future therapy.

## Highlights

• Myocardial infarction may occur without traditional cardiovascular risk factors
• Monocyte cytokine production capacity is increased in such patients
• This coincides with an enrichment of H3K4me3 at cytokine promotors
• These findings offer potential treatment targets in these patients

## Graphical abstract

**Figure:**
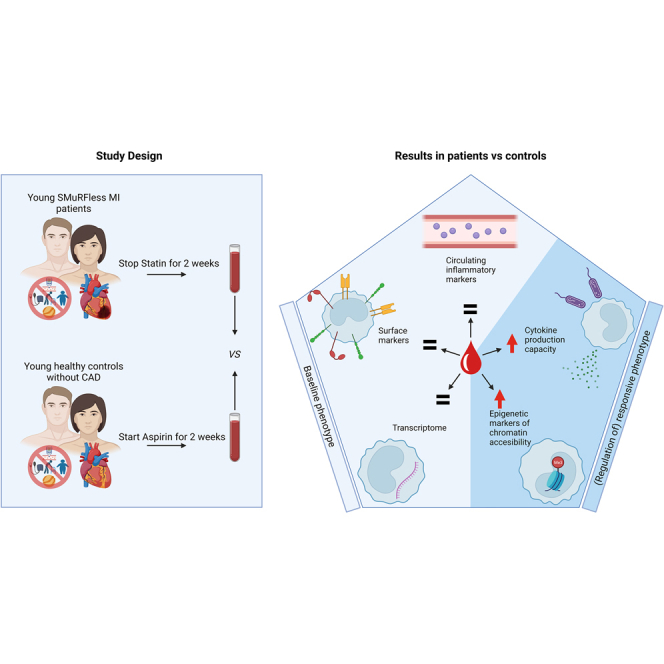

## Introduction

Atherosclerosis is a major cause of worldwide morbidity and mortality, with myocardial infarction (MI) being one of the most severe manifestations of this process.^1^ The development of atherosclerosis is associated with modifiable risk factors such as dyslipoproteinemia, hypertension, diabetes mellitus, smoking, and obesity. Over the past decades, improved control of these risk factors, e.g. with lipid-lowering therapy, has led to significant improvements in cardiovascular outcome.

There is however a significant proportion of patients that develops MI in the absence of these risk factors.^2^ Strikingly, these patients have an increased risk of in-hospital mortality compared to patients with at least one standard modifiable risk factor (SMuRF).^3^ These so called “SMuRFless” patients with MI lack the traditional targets for pharmacological intervention, even though atherosclerosis is clearly present. Therefore, unraveling the pathophysiological processes that drive the increased cardiovascular risk in this specific group of patients is imperative in order to develop more effective treatment strategies.

The concept that atherosclerosis is merely caused by the passive accumulation of cholesterol in the vessel wall has dominated the field of cardiovascular disease for many years. Nowadays the picture is much more complex with atherosclerosis being established as a chronic, low-grade inflammatory disorder of the arterial wall. The CANTOS trial was one of the first randomized controlled trials targeting inflammation in patients who suffered a previous MI and had elevated C-reactive protein (CRP) concentrations.^4^ In this landmark trial, treatment with the interleukin (IL)-1β neutralizing antibody canakinumab reduced the risk of subsequent major cardiovascular events independent of circulating lipid concentrations. More recently, the nonspecific anti-inflammatory drug colchicine has also been shown to reduce cardiovascular event rate in high-risk patients.^5^^,^^6^

Cells of the innate immune system, especially monocyte-derived macrophages, are the most abundant cells in the atherosclerotic plaque and play a pivotal role in different stages of atherosclerosis.^7^ Recently, we and others showed that monocytes can build an immunological memory, which could contribute to persistent atherosclerotic inflammation.^8^ This mechanism, termed “trained immunity”, is mediated through distinct epigenetic and metabolic reprogramming.^9^ Trained immunity can be induced by micro-organisms, but also by endogenous atherogenic stimuli, such as lipoproteins, glucose, and catecholamines.^10^^,^^11^^,^^12^^,^^13^ Trained monocytes are characterized by enhanced cytokine production capacity and enrichment of activating epigenetic histone markers such as histone 3 lysine 4 trimethylation (H3K4me3), or a reduction in repressive H3K9me3 and H3K27me3 on the corresponding cytokine genes.^14^ We have recently demonstrated the presence of these markers in patients with increased cardiovascular risk due to familial hypercholesterolemia (FH) or pheochromocytoma.^13^^,^^15^

We hypothesize that in SMuRFless patients who suffered an MI, in particular those at young age, immune cell activation and more specifically trained immunity contributes to the pathophysiology of cardiovascular disease. Therefore, we assessed circulating monocytes in SMuRFless patients with MI and matched controls without coronary artery disease. We analyzed monocyte phenotype and function, and specific histone marks associated with trained immunity.

## Results

### Baseline characteristics

The clinical characteristics of patients with MI and controls are listed in Table 1. Patients and controls were matched for age, sex, BMI, and smoking status. Additionally, there were no significant differences between subjects in blood pressure or family history of cardiovascular disease. In patients, the MI had occurred at a median of 25 (19–38) months prior to inclusion. In controls, there was a median of 28 (7–42) months between the CT-scan and inclusion. The proportion of active smokers was low in both groups, with 3 (15%) active smokers among patients and 2 (11%) among controls. Evidently, in the patients, there was significantly more use of aspirin, statins, angiotensin-converting enzyme inhibitors/angiotensin receptor blockers, and β-blockers compared to controls because of their previous MI. Statin therapy was successfully interrupted in all 20 patients with MI, and aspirin therapy was successfully completed in all 18 control participants during the 14 days prior to blood sampling. Laboratory characteristics for both groups are listed in Table 1. There were no significant differences between groups in total cholesterol, low-density lipoprotein cholesterol (LDLc), or triglycerides after 2 weeks of statin cessation. High-density lipoprotein cholesterol was slightly lower in the MI group compared to the control group (1.2 (1.0–1.4) vs. 1.4 (1.2–1.6) mmol/L (p = 0.03)). The median levels of both fasting glucose and lipoprotein(a) (Lp(a)) were not elevated, and did not significantly differ between groups. The clinical characteristics of patients with MI at the time of the index event and lipid levels before interruption of statin therapy are included in Tables S1 and S2, respectively.

**Table 1:** Clinical and laboratory characteristics

Clinical and laboratory characteristics	Patients n = 20	Controls n = 18	p value
Age, (years)	48 (46–52)	50 (46–51)	0.59
Sex, male (%)	15 (75%)	13 (72%)	0.57
Race, white (%)	20 (100%)	18 (100%)	–
MI type, STEMI (%)	13 (65%)	–	–
Time between MI and V1 (months)	25 (19–38)	–	–
Time between CT and V1 (months)	–	28 (7–42)	–
BMI (kg/m^2^)	27 (24–29)	27 (24–30)	0.92
Systolic blood pressure (mmHg)	121 (118–136)	122 (120–128)	0.70
Diastolic blood pressure (mmHg)	80 (78–83)	81 (77–89)	0.46
Family history of cardiovascular disease (%)	8 (42%)	3 (17%)	0.09
Smoking, active (%)	3 (15%)	2 (11%)	0.67
Smoking, past (%)	9 (45%)	6 (33%)	
Smoking, never (%)	8 (40%)	10 (56%)	
Acetylsalicylic acid use (%)	20 (100%)	1 (6%)	<0.0001
Statin use (%)	19 (95%)	0 (0%)	<0.0001
ACE inhibitor/ARB use (%)	15 (75%)	1 (6%)	<0.0001
β-Blocker use (%)	13 (65%)	0 (0%)	<0.0001
Ezetimibe use (%)	4 (20%)	0 (0%)	0.07
Total c (mmol/L)	5.2 (4.5–5.7)	5.5 (4.8–5.9)	0.37
LDLc (mmol/L)	3.4 (2.9–3.6)	3.4 (2.8–4.0)	0.57
HDLc (mmol/L)	1.2 (1.0–1.4)	1.4 (1.2–1.6)	0.03
Triglycerides (mmol/L)	1.4 (1.0–2.0)	1.2 (0.9–1.7)	0.38
Lipoprotein(a) (mg/dL)	12.0 (5.9–38.4)	14.1 (3.8–33.0)	0.65
Fasting glucose (mmol/L)	5.2 (4.8–5.6)	5.2 (4.9–5.3)	0.56
Creatinine (μmol/L)	81 (72–97)	82.5 (73–94)	0.96
Leukocyte count (10³/μL)	5.5 (4.9–6.9)	5.5 (4.9–6.4)	0.87
Neutrophil count (10³/μL)	3.2 (2.5–4.3)	3.0 (2.7–3.7)	0.85
Lymphocyte count (10³/μL)	1.62 (1.3–1.9)	1.7 (1.4–1.9)	0.65
Monocyte count (10³/μL)	0.5 (0.5–0.6)	0.5 (0.4–0.6)	0.67
Neutrophil percentage (% of leukocytes)	54.7 (52.3–62.4)	54.0 (51.7–59.9)	0.74
Lymphocyte percentage (% of leukocytes)	30.3 (23.4–35.5)	33.0 (25.9–35.8)	0.55
Monocyte percentage (% of leukocytes)	8.8 (7.6–9.8)	8.7 (7.7–10.3)	0.95
Neutrophil/lymphocyte ratio	1.8 (1.5–2.4)	1.6 (1.5–2.3)	0.71
Monocyte/lymphocyte ratio	0.3 (0.2–0.4)	0.3 (0.3–0.3)	0.66

Data are presented as median and interquartile range for continuous- and percentages for categorical variables. See also Tables S1 and S2*.*

(ACE) angiotensin converting enzyme, (ARB) angiotensin receptor blocker, (BMI) body mass index, (CT) computed tomography scan, (HDLc) high-density lipoprotein cholesterol, (LDLc) low-density lipoprotein cholesterol, (MI) myocardial infarction, (STEMI) ST-elevation myocardial infarction, (Total c) total cholesterol.

### Circulating markers of systemic inflammation

**Figure 1:**
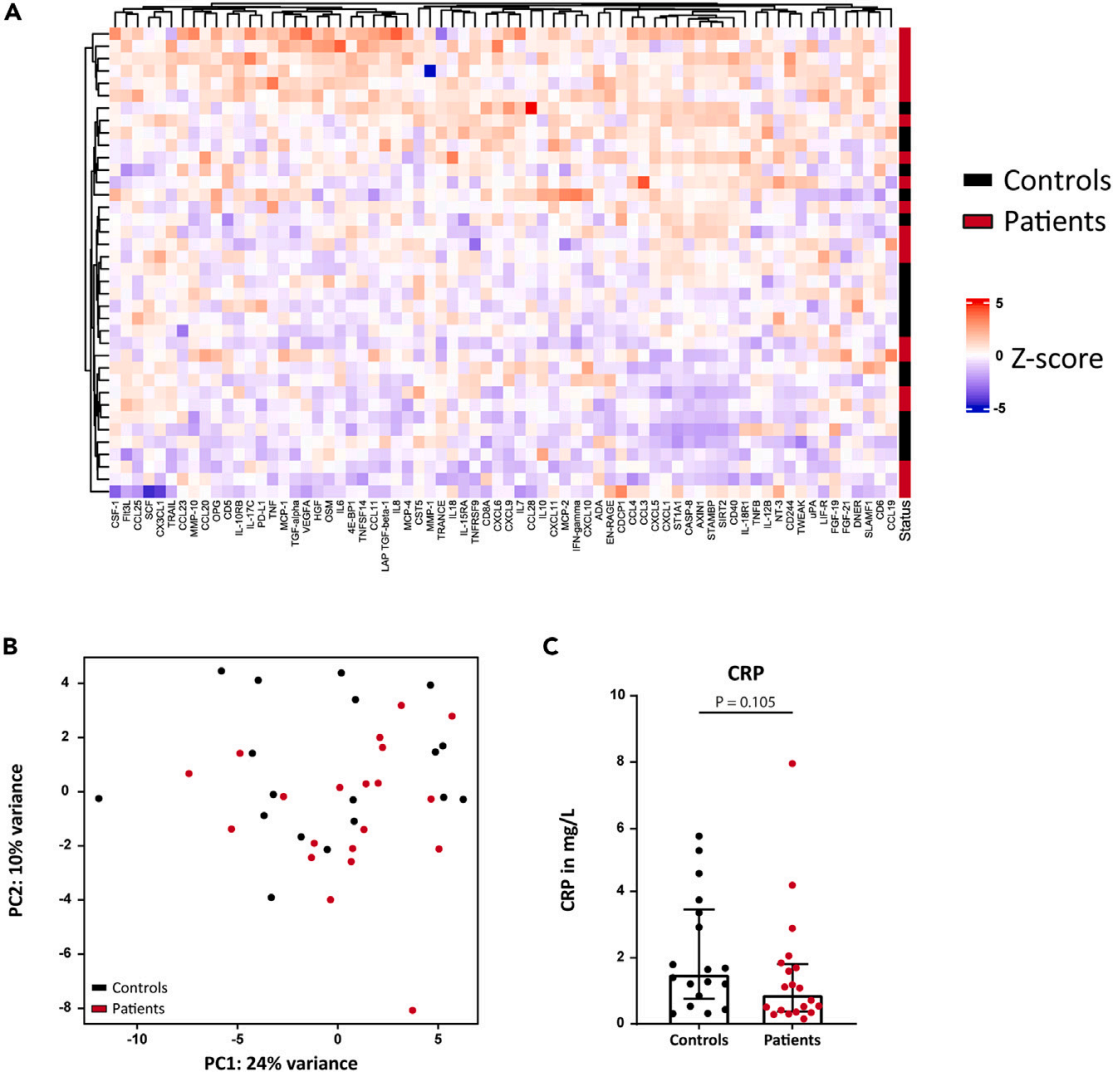
Circulating inflammatory markers Circulating inflammatory markers were measured using a targeted proteome platform. (A) A heatmap assessing each protein separately did not show discriminating markers between patients and controls. (B) PCA plot showed no clear separation of the patients and controls. A separate ELISA was performed to determine circulating CRP levels. (C) CRP levels did not differ between patients and controls. Data are presented as median with interquartile range. N = 18 controls vs. 20 patients.

A targeted proteome platform was used to measure 96 inflammatory markers in plasma samples of all subjects. Overall, there was no clear separation of the patients and controls based on circulating inflammatory markers (Figure 1B). Moreover, assessing each protein separately, there was no specific marker that was significantly higher in patients or controls (Figure 1A). Since CRP is the most widely used marker for (low grade) systemic inflammation, we performed separate ELISA measurements to determine circulating CRP levels. Notably, CRP levels did not significantly differ between patients (0.91 (0.38–1.83) mg/L) and controls (1.54 (0.77–3.49) mg/L (p = 0.11)) (Figure 1C).

### Circulating monocyte counts and subsets are similar in SMuRFless patients and controls

**Figure 2:**
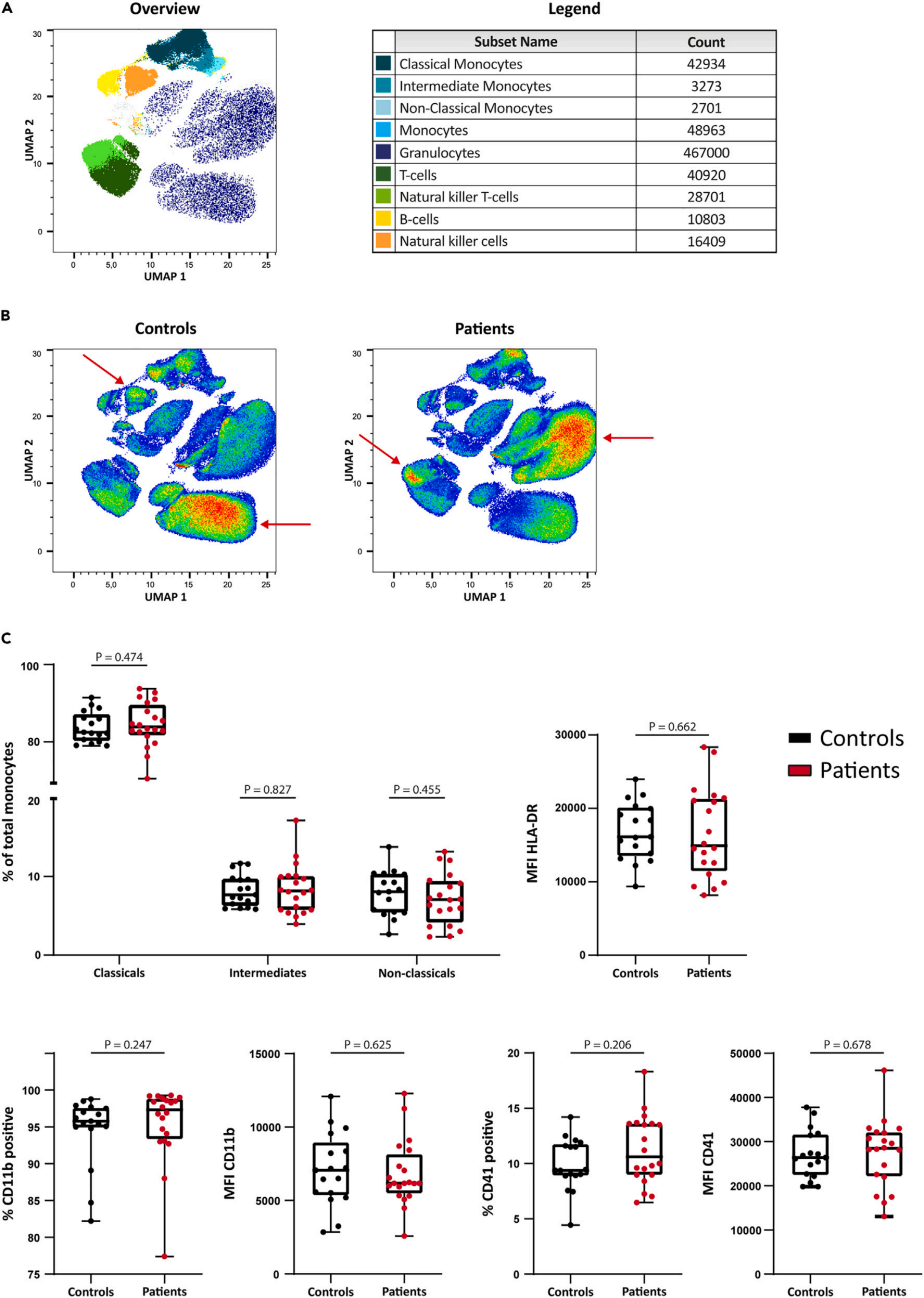
Circulating immune cells Circulating immune cells of controls and patients were analyzed using flow cytometry. (A) uMAP plot of flow cytometry results combined for controls and patients with gating for cell type. (B) uMAP plot of flow cytometry results seperated for controls and patients shows no difference in monocyte (subset) phenotypes. A difference was seen between controls and patients in specific granulocyte, natural killer, and natural killer-T cell subsets (red arrows). (C) Manual gating revealed no difference in monocyte subsets. The percentage of CD11b+ and CD41^+^ monocytes, or the MFI of HLA-DR, CD11b, and CD41 on monocytes did not differ between controls and patients. Data are presented as box and whisker plots with minimum and maximum. N = 17 controls vs. 20 patients. See also Figures S1–S3.

Circulating leukocyte counts in patients and controls are shown in Table 1. No differences were seen in absolute or relative counts of different leukocytes between patients and controls. Neutrophil-lymphocyte and monocyte-lymphocyte ratios did not differ either. Using flow cytometry, we performed a more detailed analysis of blood cell phenotype. The unsupervised uniform manifold approximation projection (uMAP) analysis of whole blood in patients and controls is shown in Figures 2A and 2B. The uMAP did not show any differences in the percentage of monocytes or monocyte subsets between patients and controls based on the cell surface expression markers. Monocytes and monocyte subset populations were also studied in more detail using manual gating. No differences were seen in the percentage of classical, intermediate, or nonclassical monocytes (Figure 2C). Moreover, there was no difference in the percentage of monocytes that expressed CD11b or CD41. The median fluorescent intensity of CD11b, CD41, and HLA-DR did not differ either (Figure 2C).

Within other leukocyte populations, uMAP analysis demonstrated differences within the granulocytes, natural killer (NK) cells, and NK-T cells between patients and controls (Figure 2B). Further preliminary assessment of the granulocyte population revealed an upregulation of the activation marker CD16 (p = 0.013) on neutrophils from patients compared to controls. Although HLA-DR expression was also numerically higher in patients, this difference was not statistically significant (p = 0.081). CD11b expression was similar in both groups (Figure S3).

### Monocytes from SMuRFless patients with MI have a similar transcriptomic profile at baseline compared to controls

**Figure 3:**
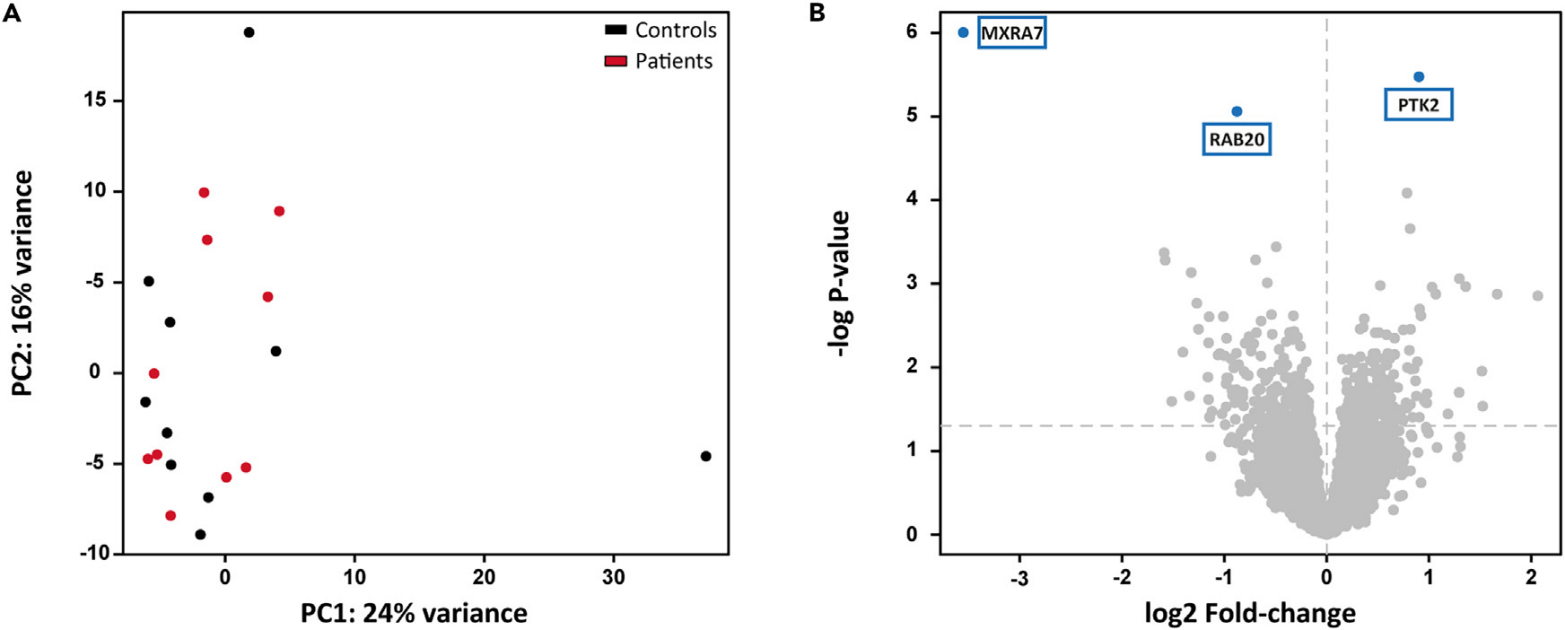
Monocyte baseline transcriptome Differential expression analysis was performed after RNA sequencing of unstimulated monocytes isolated from controls and patients. (A) PCA plot of the filtered result (≥10 counts in more than 75% samples resulting in 12185 genes) which does not show differences in the primary components that separate patients from controls. (B) Volcano plot of DE genes highlighting the 3 genes that are significantly differentially expressed after false discovery correction. N = 10 controls vs. 10 patients. See also Figure S5.

RNA sequencing of circulating unstimulated monocytes showed that there were no patterns of expression that distinguish patients from controls at the genome-wide level. Principal component analysis (PCA) of all expressed genes did not separate the groups in any of the components (Figure 3A). We subsequently performed PCA with only those genes identified as being related to inflammation according to GO term GO:0006954 and also this did not separate patients from controls (Figure S5). Differential expression analysis identified only three genes that were significant following multiple testing correction (FDR ≤0.05): MXRA7 (p_adj_ = 0.012), and RAB20 (p_adj_ = 0.035) were significantly lower expressed in patients, whereas PTK2 (p_adj_ = 0.02) was higher expressed (Figure 3B). Pathway analysis of the 100 most up- or downregulated genes using Reactome^16^ or WikiPathways^17^ did not identify any significantly enriched pathways after multiple testing correction.

### PBMCs isolated from SMuRFLess patients with MI show enhanced cytokine production capacity

**Figure 4:**
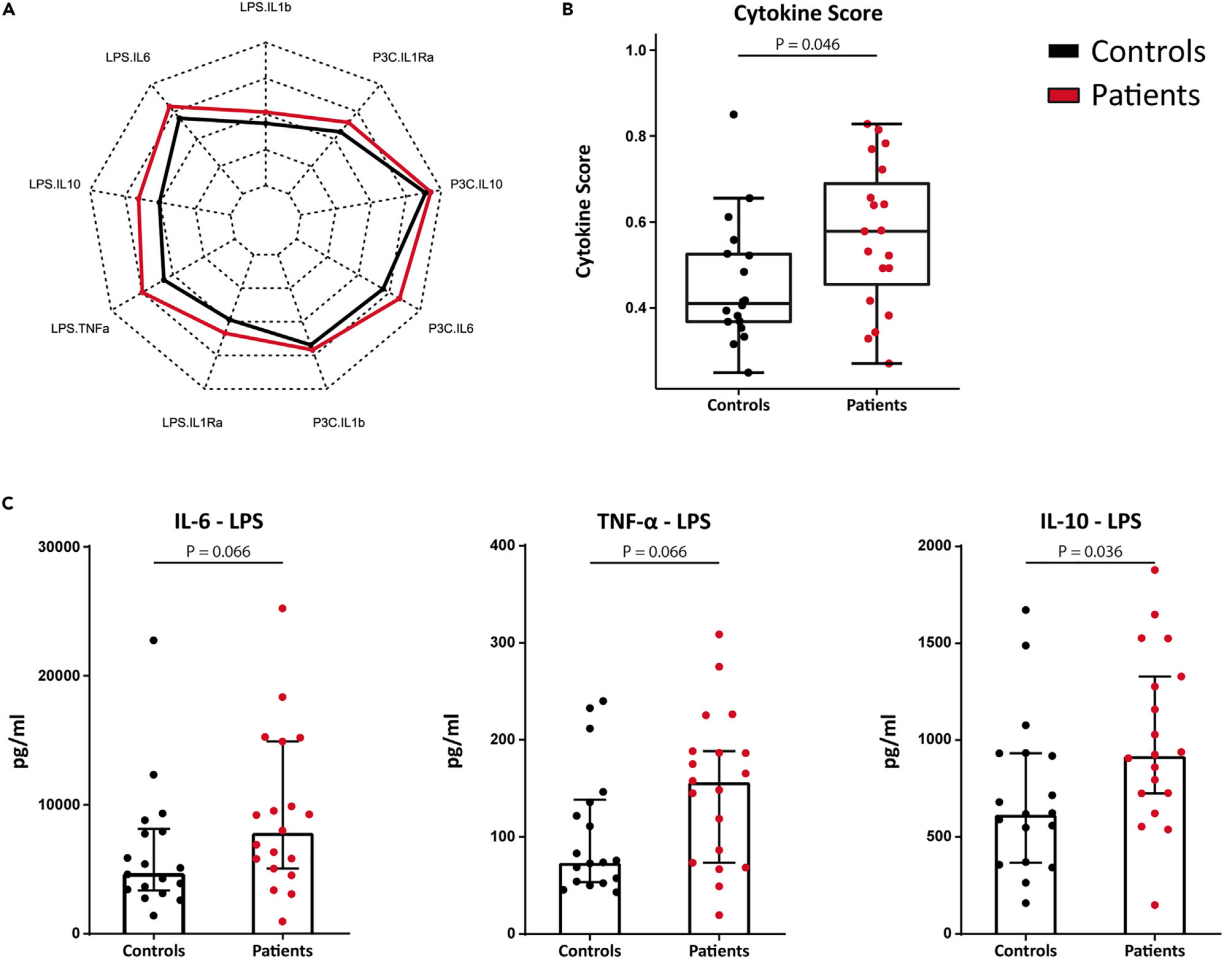
PBMC cytokine production capacity PBMCs isolated from controls and patients were stimulated with TLR agonists LPS and P3C for 24 h before measuring cytokine production. (A) A radar plot shows the mean cytokine production by controls and patients for each stimulation and cytokine. This reveals that the difference between groups is mainly driven by enhanced production of TNF-α, IL-6, and IL-10 upon LPS stimulation. (B) A combined cytokine score reveals an overall elevated cytokine production in patient compared to controls. Data are presented as box and whisker plots with 95% CI. (C) Shows production of TNF-α, IL-6, and IL-10 upon LPS stimulation in more detail. Data are presented as median with interquartile range. N = 18 controls vs. 19 patients. See also Figure S4 and Table S3.

To investigate whether peripheral blood mononuclear cells (PBMCs) isolated from SMuRFless patients with MI are functionally different from controls, we stimulated PBMCs *ex vivo* with Toll-like receptor 4 and 2 ligands (lipopolysaccharide (LPS) and Pam3Cys) for 24 h and measured cytokine production of TNF-α, IL-6, IL-1β, IL-1Ra, and IL-10. The concentrations of all individual cytokines we measured were consistently numerically higher in patients versus controls (Figure 4A). However, most cytokines did not reach statistical significance on their own (Figure S4). We summarized these cytokine values in one cytokine score, that was previously established,^18^ by using principal component analysis as dimensional reduction. We extracted the loadings of PC1 and PC2 which captured 72% of the variance in the data (inclusion of more components did not change the results) for logistic regression. This model then established that there is a statistically significant difference across all cytokine production experiments that distinguishes these patients from matched controls (p = 0.046, Figure 4B). For most study participants, TNF-α concentrations upon P3C stimulation were below the detection limit and were therefore not included in the cytokine score. Since the difference in the cytokine score appeared to be mainly driven by an enhanced production of TNF-α, IL-6, and IL-10 after LPS stimulation (Figures 4A and 4C), these cytokine genes were selected for further examination of specific histone marks associated with trained immunity.^14^

In a previous study in patients with very high LDLc concentrations due to familial hypercholesterolemia, we have reported increased cytokine production capacity compared to normocholesterolemic controls.^15^ To explore whether the cytokine production capacity in the study groups was affected by plasma cholesterol levels, we calculated correlations between LDLc concentrations (two weeks after stopping statins) and the cytokine production after stimulation with LPS or Pam3Cys using Spearman correlation. The results are shown in Table S3. A significant positive correlation in the patient group was only found for IL-6 after Pam3Cys stimulation (Spearman r 0.56, p = 0.02) and in the control group for IL-1β after Pam3Cys stimulation (Spearman r 0.56, p = 0.02). Therefore, the circulating LDLc concentrations do not seem to contribute to the differences we observed in cytokine production capacity between the patients and controls.

### Hyperresponsiveness in monocytes from SMuRFless patients with MI is associated with more transcriptionally permissive H3K4me3 on cytokine gene promoter

**Figure 5:**
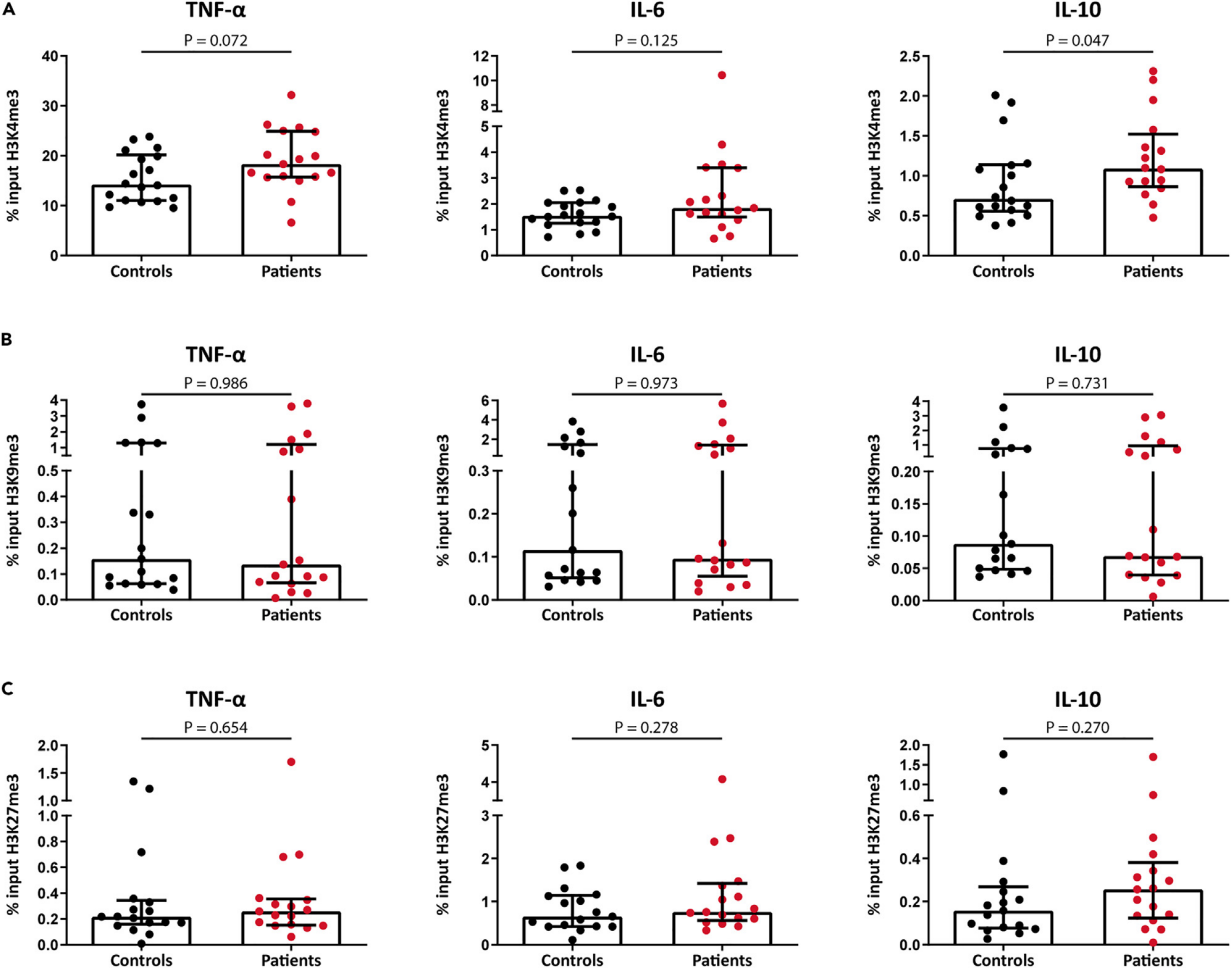
Histone modifications on cytokine gene promoters ChIP-qPCR analysis was performed on unstimulated monocytes isolated from controls and patients. ChIP was performed for H3K4me3 (A), H3K9me3 (B), and H3K27me3 (C) on the promoter regions of *TNF-α, IL-6*, *and IL-10*. (A) H3K4me3 was significantly increased on the promoter region of *IL-10* in patients versus controls. For *TNF-α* and *IL-6*, H3K4me3 levels did not reach statistical significance. There were no differences in the repressive markers H3K9me3 and H3K27me3. (B and C) Data are presented as median with interquartile range. N = 18 controls vs. 17 patients for H3K4me3, and N = 17 controls vs. 17 patients for H3K9me3 and H3K27me3. In IL-10 H3K4me3, one outlier in the patient group was removed from analysis.

In order to investigate whether the hyperresponsive phenotype of the patients is due to trained immunity, we explored the histone markers most often involved; the transcriptionally permissive H3K4me3 and the transcriptionally repressive H3K9me3 and H3K27me3. We performed ChIP-PCR for these epigenetic markers on the promoter regions of the genes encoding TNF-α, IL-6, and IL-10 (the cytokines most markedly upregulated in patients after *ex vivo* stimulation) (Figure 5). Levels of H3K4me3 on *IL-10* were significantly higher in patients versus controls (p = 0.04). For *TNF-α* and *IL-6*, H3K4me3 levels did not reach statistical significance. There were no differences in the repressive markers H3K9me3 and H3K27me3 on the promoters of any of the cytokines between groups.

## Discussion

An increasing number of patients suffer from myocardial infarction in the absence of traditional cardiovascular risk factors.^2^ Identifying the underlying mechanisms of coronary artery disease in these patients is paramount in order to develop more targeted treatment strategies. In the current study, we show that SMuRFless patients suffering an MI at a young age are characterized by a hyperresponsive state of innate immune cells. This is likely mediated via increased chromatin accessibility at the promoters of inflammatory genes. Notably, this is present without further indications of systemic inflammation, such as increased CRP levels, or widespread baseline transcriptional differences in monocytes. These data suggest that inflammatory reprogramming of circulating monocytes in a process called trained immunity contributes to MI in young SMuRFless patients and offers potential targets for pharmacological prevention.

Monocyte-derived macrophages are the most important immune cells in atherosclerotic plaques and are key in the pathophysiology of MI. There is accumulating evidence that long-term epigenetic monocyte reprogramming, termed trained immunity, contributes to atherogenesis.^19^ After transient exposure to pathogens or damage-associated molecular patterns, monocytes and macrophages mount an enhanced pro-inflammatory response upon subsequent encounters with similar or unrelated stimuli. This trained immune phenotype is mediated via distinct epigenetic and metabolic reprogramming of these cells.^9^ In the context of cardiovascular disease (CVD), it has previously been shown that pro-atherogenic particles such as oxidized low-density lipoprotein (oxLDL) can induce trained immunity via enrichment of H3K4me3 at the level of pro-inflammatory cytokine genes.^10^ This epigenetic phenotype is also seen in patients with an increased CVD risk due to FH or pheochromocytoma.^13^^,^^15^ Christ et al. showed that in *Ldlr*^−/−^ mice, a Western-type diet induced this functional immune cell reprogramming along with systemic inflammation.^20^ Interestingly, after switching back to chow diet, the systemic inflammation subsided whereas the myeloid cell responses toward innate immune stimuli remained augmented. This persistent change was mediated by transcriptional and epigenetic reprogramming at the level of hematopoietic stem and progenitor cells.

In the current study, *ex vivo* stimulated monocytes from SMuRFless patients with MI mounted an exaggerated overall cytokine response compared to their matched controls. Analysis of the individual cytokines revealed a higher IL-10 production after LPS stimulation, and a trend for TNF and IL-6 (p = 0.066). The enhanced cytokine production capacity of these cells was associated with a difference in the histone modification at the level of the promoter of cytokine genes, which is characteristic of trained immunity. Patients showed an enrichment of the activating histone methylation marker H3K4me3 on the promoter of IL-10, whereas no differences were found in the repressive markers H3K9me3 and H3K27me3. Although H3K4me3 is the most classical histone marker implicated in trained immunity, many other histone markers and epigenetic processes are involved in shaping overall chromatin accessibility in trained immunity.^21^ Consistent with our findings, patients with FH and patients with pheochromocytoma with increased risk of CVD are also characterized by a hyperresponsive monocyte phenotype, which coincides with H3K4me3 enrichment.^13^^,^^15^ However, in a previous study in patients with traditional risk factors and established coronary atherosclerosis, the augmented cytokine production was accompanied by a lower H3K4me3 presence, but also a lower presence of the repressive histone modification H3K27me3.^22^ Furthermore, mirroring the findings in the study by Christ et al., this trained immune phenotype was not accompanied by signs of systemic inflammation such as increased levels of CRP or other circulating inflammatory markers.

Although monocytes from SMuRFless patients with MI were hyperresponsive upon stimulation, unstimulated monocytes showed no differences in surface activation markers or transcriptome apart from three genes. This is consistent with the phenotype of trained monocytes, that have returned to their baseline homeostatic state, but are primed to behave hyperresponsive after restimulation. Three genes were differentially expressed in the unstimulated condition. Protein tyrosine kinase 2, a protein involved in cellular adhesion and previously described as possible therapeutic target against atherosclerosis,^23^ was upregulated in SMuRFless patients with MI, whereas Ras-related protein RAB20 and Matrix Remodeling associated 7 (MXRA7) were downregulated. RAB20 is known to be involved in brain inflammation^24^ and a lower expression has been described in patients with obstructive sleep apnea.^25^ The MXRA family of genes has been suggested to be involved in the pathophysiology of psoriasis and inflammatory liver disease.^26^ Although both genes evidently play a role in inflammatory diseases, to date neither of these genes have been described as being involved in CVD.

Our findings illustrate that while the monocytes from SMuRFless patients with MI seem phenotypically indistinctive from controls in resting state, they are functionally primed to evoke a more aggressive response when triggered. This could, for instance, take place in the circulation or after migration into the atherosclerotic plaque, and thereby contribute to the progression of atherosclerosis. Nevertheless, it remains unknown what stimulus could have induced the trained immune state in these patients in the absence of traditional pro-atherogenic triggers. *In vitro*, trained immunity can be induced by various endogenous atherogenic molecules such as oxLDL, Lp(a), catecholamines, glucose, and aldosterone.^8^ We did not assess stress exposure or perceived stress and related catecholamine exposure in our patients, nor did we measure aldosterone. In addition, training can be induced by previous infections or vaccinations, and it has been proposed as potential mechanism that links the infectious burden with cardiovascular disease prevalence.^27^ We did not assess previous infections in our patients. Furthermore, in the patients included in this study, the MI had occurred more than 1 year before blood sampling. This timing was chosen in order to mitigate any acute systemic effects that are known to arise after MI.^28^ Although this does not completely rule out any possible long-term effect of the MI itself, it was previously shown that in Bacillus Calmette-Guérin vaccination-induced trained immunity, the enhanced cytokine production capacity of innate immune cells returned back to baseline after 1 year^29^ Finally, there is a large inter-individual variation in the response to stimuli that can induce trained immunity, which is associated with single-nucleotide polymorphisms in various pathways known to be important in this process.^30^^,^^31^ One may hypothesize that SMuRFless patients with MI are particularly susceptible to stimuli that would normally not be capable of inducing trained immunity.

The strength of this study is the extensive phenotyping of circulating monocytes in a well characterized and matched group of patients: patients that suffered an MI before the age of 50 without SMuRFs that were carefully matched to control subjects without coronary atherosclerosis. In addition, statins and aspirin use was matched for two weeks. We specifically assessed patients who had undergone an MI at the age of 50 years or younger, as advanced age itself is associated with chronic low-grade inflammation.^32^

### Limitations of the study

Our study also has a couple of limitations. First, a relatively liberal LDLc cutoff of 4.5 mmol/L (95% percentile of normal distribution) was used to indicate dyslipoproteinemia, but there was no difference in LDLc between patients and controls (3.4 mmol/L in both). Second, we found slightly lower concentrations of HDLc in patients compared to controls, although still in the normal range, which could have potentially contributed to the differences in immune cell function. Third, as a result of the explorative nature of this study, no formal sample size calculation was performed and the sample size was relatively small. However, we have previously shown significant differences in cytokine production capacity and epigenetic markers of trained immunity in studies with comparable group sizes.^13^^,^^22^ Nonetheless, it is important to validate these results in a larger group of SMuRFless patients with MI. Fourth, since we only included five female participants in both groups, no analysis on sex-differences could be performed. Fifth, we only measured three specific epigenetic markers of trained immunity on the promotor regions of specific cytokine genes, whereas the overall chromatic accessibility is defined by more epigenetic processes, which might be different for different stimuli. Future studies should therefore include a more comprehensive epigenetic evaluation. Last, as we already assessed absolute leukocyte counts using a Sysmex hematology analyzer, we decided not to include count-beads in the flow cytometry measurements, hence limiting interpretation of absolute counts in this specific assay. Moreover, in this study, we mainly focused on characterization of monocytes, as monocyte-derived macrophages are the most abundant immune cells in atherosclerotic plaques. Yet, the contribution of other immune cells should also be investigated in the context of the SMuRFless MI patient group. In the uMAP flow cytometry analysis, we identified a difference in granulocyte subset between patients and controls. Further preliminary analyses indeed showed upregulated expression of CD16 and HLA-DR on the neutrophils of patients compared to controls. Unfortunately, due to the monocyte-focused design of our flow cytometry panel, we could not perform a deeper analysis of these cells. This does offer an interesting new target for further study, as neutrophils can also adopt a trained phenotype,^33^ and their involvement in atherosclerosis has recently gained considerable interest.^34^^,^^35^

In conclusion, our study shows that circulating monocytes in SMuRFless patients with MI are characterized by a hyperresponsive phenotype. This behavior is associated with enrichment of a permissive histone marker at the promoter region of key inflammatory genes. This is indicative of trained immunity and it was observed in the absence of active systemic inflammation. Considering the lack of intervenable risk factors in this specific patient group, this could be a promising target for future therapy.

## STAR★Methods

### Key resources table

**Table:** 

REAGENT or RESOURCE	SOURCE	IDENTIFIER
**Antibodies**		

Anti-human CD45 KromeOrange, Clone J33	Beckman Coulter	Cat# A96416, RRID:AB_2888654
Anti-human HLA-DR PE, Clone immu-357	Beckman Coulter	Cat# IM1638U, RRID:AB_2876782
Anti-human CD14 PECy7, Clone 61D3	eBioscience	Cat# 25-0149-42, RRID:AB_1582276
Anti-human CD16 FITC, Clone CD16	eBioscience	Cat#: 11-0168-42, RRID:AB_10805747
Anti-human CD3 APC-Alexa750, Clone UCTH1	Beckman Coulter	Cat# A66329, RRID:AB_2876783
Anti-human CD56 APC, Clone N901	Beckman Coulter	Cat# IM2474U, RRID:AB_2876784
Anti-human CD192 (CCR2) BV421, Clone 48607	Becton Dickinson	Cat# 564067, RRID:AB_2738573
Anti-human CD11b BV785, Clone ICRF44	Biolegend	Cat# 301346, RRID:AB_2563794
Anti-human CD41 PerCP/Cy5.5, Clone Hip8	Biolegend	Cat# 303719, RRID:AB_2561731
Anti-human H3K4me3 (pAb-003-050)	Diagenode	Cat# C15410003-50, RRID: AB_2616052
Anti-human H3K9me3 (pAb-193-050)	Diagenode	Cat# C15410193-50, RRID: AB_2616044
Anti-human H3K27me3 (pAb-069-050)	Diagenode	Cat# C15410195 , RRID: AB_2753161

**Biological samples**		

Peripheral blood through venous puncture, 18-50 year old males and females	Homo Sapiens	Medical Ethics Committee of the Region Arnhem-Nijmegen ethical approval number NL61543.091.17

**Chemicals, peptides, and recombinant proteins**		

Pharm Lyse lysing buffer	BD Biosciences	Cat# 555899
Glutamine, 2 mmol/L in RPMI	Invitrogen	Cat# 25030081
Gentamycin, 10 mg/ml in RPMI	Centrafarm, Etten Leur, The Netherlands	N/A
Pyruvate, 1 mM in RPMI	Invitrogen	Cat# 11360070
Lipopolysaccharide from *Escherichia coli*	Sigma-Aldrich, Serotype 055:B5	Cat# L2880
Pam3CysK4	EMC Microcollections	Cat# L2000
Ficoll-Paque PLUS density gradient media	Cytiva Life Sciences, Marlborough, USA	Cat# 17144003
Roswell Park Memorial Institute (RPMI) 1640 Dutch-modified culture medium	Life Technologies/Invitrogen	Cat# 22409015
16% methanol-free formaldehyde	Thermo Fisher, Waltham, USA	Cat# 28908
Glycine	Scharlab	Cat# AC04021000
TRIzol reagent	Thermo Fisher, Waltham, USA	Cat# 15596018
Dyna beads protein A/G	Invitrogen	Cat# 10001D and Cat# 10003D
Complete Protease Inhibitor Cocktail	Merck	Cat# 11697498001
Proteinase K	Qiagen	Cat# 19131
Power SYBR Green Master Mix	Applied Biosciences	Cat# 4368708

**Critical commercial assays**		

hsCRP ELISA	R&Dsystems	Cat# DY1707, RRID:AB_2928088
Human Duoset IL-6 ELISA	R&Dsystems	Cat# DY206, RRID:AB_2814717
Human Duoset TNF ELISA	R&Dsystems	Cat# DY210, RRID:AB_2848160
Human Duoset IL-10 ELISA	R&Dsystems	Cat# DY217B, RRID:AB_2927688
Human Duoset IL-1b ELISA	R&Dsystems	Cat# DY201, RRID:AB_2848158
Human Duoset IL-1Ra ELISA	R&Dsystems	Cat# DY280, RRID:AB_2934302
RNEasy mini kit	Qiagen	Cat# 74104
QiaQuick MinElute PCR purification kit (including buffer EB)	Qiagen	Cat# 28106
Anti-human CD14 magnetic beads	Miltenyi Biotec	Cat# 130-050-201
Target 96 Inflammation panel Proximity Extension Assay	Olink Proteomics (Uppsala, Sweden)	N/A

**Deposited data**		

RNA-seq data	This paper	GEO: GSE229044

**Oligonucleotides**		

MYO forward AGCATGGTGCCACTGTGCT	Biolegio	Primer for ChIP-PCR
MYO reverse GGCTTAATCTCTGCCTCATGAT	Biolegio	Primer for ChIP-PCR
GAPDH forward CACCGTCAAGGCTGAGAACG	Biolegio	Primer for ChIP-PCR
GAPDH reverse ATACCCAAGGGAGCCACACC	Biolegio	Primer for ChIP-PCR
ZNF UTR forward AAGCACTTTGACAACCGTGA	Biolegio	Primer for ChIP-PCR
ZNF UTR reverse GGAGGAATTTTGTGGAGCAA	Biolegio	Primer for ChIP-PCR
TNFa forward CAGGCAGGTTCTCTTCCTCT	Biolegio	Primer for ChIP-PCR
TNFa reverse GCTTTCAGTGCTCATGGTGT	Biolegio	Primer for ChIP-PCR
MYT1 forward TTGGGGTTTGGAATTCTCTG	Biolegio	Primer for ChIP-PCR
MYT1 reverse CAGTCTTTGTTCTCCGCACA	Biolegio	Primer for ChIP-PCR
IL-6 forward AGGGAGAGCCAGAACACAGA	Biolegio	Primer for ChIP-PCR
IL-6 reverse GAGTTTCCTCTGACTCCATCG	Biolegio	Primer for ChIP-PCR
IL-10 forward GGGACAGCTGAAGAGGTGGA	Biolegio	Primer for ChIP-PCR
IL-10 reverse CCTCAAAGTTCCCAAGGAGC	Biolegio	Primer for ChIP-PCR

**Software and algorithms**		

FlowJo v10.8	BD Biosciences	https://www.flowjo.com/solutions/flowjo/downloads
Graphpad Prism v9.0	Graphpad software, La Jolla, California	https://www.graphpad.com/features
Trim Galore! v0.4.5	Babaham Bioinformatics, Krueger F et al.^36^	https://www.bioinformatics.babraham.ac.uk/projects/trim_galore/
Star v2.7.5a	Dobin A, et al.^37^	http://code.google.com/p/rna-star/
HTSeq-count tool v0.11.0	Anders S, et al.^38^	N/A
DESeq2	Love MI, et al.^39^	https://bioconductor.org/packages/release/bioc/html/DESeq2.html
R 4.2.2	R Studio^40^	https://www.r-studio.com/
SPSS v25	SPSS Statistics, IBM Corp, Armonk, USA	https://www.ibm.com/products/spss-statistics
GRCh38.95 human genome	Ensembl	N/A

**Other**		

CytoFLEX flow cytometer	Beckman Coulter	N/A
BGI DNBSeq400 platform	Beijing Genomics Institute	N/A
Finger Stick Freestyle Precision blood glucose test	Abbot Laboratories, Abbot Park, USA	Cat# 7157975
Sysmex-XN 450 hematology analyzer	Sysmex, Kobe, Japan	N/A
Bioruptor Pico sonicator	Diagenode, Denville, USA	Cat#B01060010

### Resource availability

#### Lead contact

Further information and requests for resources and reagents should be directed to and will be fulfilled by the lead contact, dr. Saloua El Messaoudi (Saloua.Elmessaoudi@radboudumc.nl).

#### Materials availability

This study did not generate new unique reagents.

### Experimental model and study participant details

#### Ethical committee approval

This study was conducted at the Radboudumc and the Canisius Wilhelmina Hospital in Nijmegen, the Netherlands. The study was approved by the Medical Ethics Committee of the Region Arnhem-Nijmegen under file number NL61543.091.17, and was conducted in accordance with the ethical standards laid down in the 1964 Declaration of Helsinki and its later amendments. All participants gave informed consent prior to inclusion in the study.

#### Patient population

This study was designed as an exploratory study, hence no formal sample size calculation was performed. However, we have previously shown significant differences in cytokine production capacity and epigenetic markers of trained immunity in groups of comparable size. For example, statistically significant differences were found in 10 pheochromocytoma patients versus 14 essential hypertension patients,^13^ as well as in 20 patients with symptomatic coronary atherosclerosis versus 20 control subjects.^22^

Patients who presented with a type 1 myocardial infarction (MI) caused by atherothrombotic coronary artery disease more than 1 year and less than 4 years prior to the current study were screened for eligibility. This timing was chosen in order to avoid any acute systemic effects of the MI. To be eligible, patients had to be 50 years or younger and have none of the following standard modifiable risk factors (SMuRFs) at the time of MI: diabetes mellitus, hypertension, dyslipoproteinemia (defined as a low-density lipoprotein cholesterol (LDLc) > 4.5 mmol/L), or obesity (defined as BMI > 30 kg/m^2^). Smoking was not considered a strict exclusion criterium, as it has previously been shown that it does not affect circulating inflammatory markers and peripheral blood mononuclear cell (PBMC) cytokine production capacity, and it was carefully matched in the control group.^41^ Additionally, patients had no history of a coagulation- or inflammatory disorder, did not use any anti-inflammatory drugs at the time of blood sampling, and did not have a recent history (within 1 month) of infection or vaccination.

Subjects who had an Agatston score of 0 on a coronary computed tomography (coronary CT-scan) within the last 5 years were screened for eligibility as controls. Control participants were matched to MI patients based on sex assigned at birth, age, BMI, and smoking status.

After screening for eligibility 20 patients and 18 control subjects were included. All of the patients and control subjects completed the study. The baseline characteristics (i.e. age, sex and race) can be found in Table 1.

### Method details

#### Blood sampling

Venous blood samples were collected from 20 young SMuRFLess MI patients and 18 matched controls without coronary atherosclerosis. The blood samples from MI patients were obtained after 14 days of interruption of statin treatment, as statins potentially have immunomodulating effects.^42^ After 4-5 days, all statins have been completely cleared from the circulation. Circulating markers of inflammation were previously found to increase to pre-statin therapy concentrations within days after interrupting statins.^43^^,^^44^ We extended this period to two weeks since this would allow also cholesterol levels to rise towards pre-statin concentrations. Since aspirin can also affect cytokine production, matched controls received aspirin 80 mg once daily for 14 days, and blood samples were taken after this regimen.^45^ Consequently, both groups were all on the same dose of aspirin and were free of statin treatment at the moment of final blood sampling.

#### Cardiovascular and anthropometric assessment

Physical examination of patients and controls was performed at baseline. Information on height and weight were obtained to calculate the BMI (kg/m2). Heart rate and blood pressure were measured with a manual sphygmomanometer during rest in a supine position. Capillary fasting blood glucose levels were assessed using a finger stick (Abbott Laboratories, Abbott Park, USA). Venous EDTA and serum blood samples were centrifuged for 10 minutes at 3800 rpm at room temperature (RT). Plasma and serum were removed and immediately stored at -80°C until analysis. Total cholesterol (TChol), high-density lipoprotein cholesterol (HDLc), triglycerides (TG), lipoprotein(a) (Lp(a)) and creatinine were measured by the hospital laboratory (Radboudumc, Nijmegen, the Netherlands) using standardized methods. LDLc was calculated with the Friedewald formula.

#### Circulating inflammatory markers

Plasma CRP concentrations were measured with a commercial high-sensitive CRP enzyme linked immune assay (ELISA) kit (R&D systems, Minneapolis, USA). In addition, targeted proteomic analysis was performed on patient and control plasma to determine the levels of 96 inflammatory proteins using the Olink Target 96 Inflammation panel (v.3022). This multiplexed proteomics panel utilizes proximity extension assay (PEA) technology (Olink Proteomics, Uppsala, Sweden), which uses paired antibodies with DNA reporter sequences that then allow amplification and quantification though RT-qPCR.^46^

#### Flow cytometry

In order to phenotype circulating immune cells, 50 μL of fresh whole blood from EDTA tubes was used for flow cytometry analysis. Blood was stained with monoclonal antibodies targeting CD45 (anti-human CD45 KromeOrange, Clone J33, Beckman Coulter), CD3 (anti-human CD3 APC-Alexa750, clone UCTH1, Beckman Coulter), CD56 (anti-human CD56 APC, Clone N901, Beckman Coulter), CD16 (anti-human CD16 FITC, Clone CD16, eBioscience), CD14 (anti-human CD14 PECy7, Clone 61D3, eBioscience), HLA-DR (anti-human HLA-DR PE, Clone immu-357, Beckman Coulter), CD11b (anti-human CD11b BV785, Clone ICRF44, Biolegend), CCR2 (anti-human CD192 (CCR2) BV421, Clone 48607, Becton Dickinson) and CD41 (anti-human CD41 PerCP/Cy5.5, Clone Hip8, Biolegend). Lysis buffer (BD Pharm Lyse Lysing buffer, BD, Franklin Lakes, USA) was added for lysis of erythrocytes. Cell populations and expression of markers were measured using a CytoFlex cytometer (Beckman Coulter, Brea, USA) which underwent daily quality control to correct for variation in laser settings. Flow cytometry data was both analyzed through unsupervised learning with uniform manifold approximation and projection (uMAP) and by manual gating with FlowJo (BD Biosciences) software (Version 10.8). First, all FCS files underwent preprocessing: debris and doublets were removed and CD45^+^ cells were selected. For unsupervised analysis, all preprocessed patient and control files were grouped using keywords and concatenated into 1 file, resulting in a total of 635000 events. uMAP was performed on the concatenated file using FLowJo v10.8 plugin according to the manufacturer’s instructions with all compensated values. All cell types were successfully separated and the corresponding density plots for each marker are shown in Figure S1. Manual gating was then used to define the clusters as described below. Gates were defined using the fluorescence-minus-one (FMO) method and compensation was performed using compensation beads.^47^^,^^48^ FMO analysis revealed that the signal of the CCR2 antibody had significant overlap with autofluorescence, which was therefore excluded from further analysis. For manual gating (Figure S2) CD45^+^ single cells were further defined into a monocyte and lymphocyte gate based on forward- and side scatter. CD3^+^ T-lymphocytes and CD56^+^ natural killer cells were subsequently excluded. Monocytes were then identified as HLA^-^DR^+^CD14^+^CD16^+^ cells. Monocyte subsets were identified on the basis of CD14 and CD16 expression (classical CD14^++^CD16^-^, intermediate CD14^++^CD16^+^, and non-classical CD14^+^CD16^++^ monocytes).^47^^,^^48^ The expression of monocyte activation markers HLA-DR and CD11b, and CD41, a marker for monocyte-platelet interaction, were examined on all monocytes. After visual analysis of unbiased flow cytometry, preliminary assessment of the granulocyte population was performed using FSC/SSC, CD16 (to gate out eosinophils) and expression of CD16, CD11b and HLA-DR as activation markers was assessed (see Figure S2 for gating strategy).

#### PBMC and monocyte isolation, processing and stimulation

PBMCs were isolated from EDTA whole blood by Ficoll density gradient centrifugation (Cytiva Life Sciences, Marlborough, USA) and resuspended in Roswell Park Memorial Institute 1640 Dutch-modified culture medium (RPMI) (Life Technologies/Invitrogen, Carlsbad, USA) supplemented with 10 mg/mL gentamicin (Centrafarm, Breda, the Netherlands), 1 mmol/L pyruvate (Invitrogen, Waltham, USA) and 2 mmol/L glutamine (Invitrogen). The cell counts and composition of the PBMC fraction were assessed by the Sysmex-XN 450 hematology analyzer (Sysmex, Kobe, Japan). To evaluate cytokine production capacity, 5×105 PBMCs per well were stimulated for 24 hours in round-bottom 96-well plates (Corning, Corning, USA) with the following stimuli: RPMI, 10 ng/mL lipopolysaccharide (LPS) purified from Escherichia coli (Sigma-Aldrich, Serotype 055:B5) via phenol re-extraction,^49^ and 10 μg/mL Pam3CysK4 (P3C) (L2000, EMC Microcollections). After 24 hours, plates were centrifuged and the supernatant of stimulated cells was collected and stored at -80°C until measurement of TNF-α, IL-1β, IL-1RA, IL-6, and IL-10 using commercially available DuoSet ELISA kits (R&D Systems).

Part of the PBMCs were used for monocyte isolation with MACS® cell separation columns using anti-human CD14 magnetic beads, according to the manufacturer’s instruction (Miltenyi Biotec, Bergisch Gladbach, Germany). One million CD14^+^ monocytes were subsequently stored at -80°C after addition of TRIzol Reagent (Thermo Fisher, Waltham, USA) for later RNA sequencing. For chromatin immunoprecipitation polymerase chain reaction (ChIP-PCR), at least three million monocytes were fixed with 1% methanol-free formaldehyde (Thermo Fisher, Waltham, USA) for 10 minutes and quenched with glycine (Scharlau) 1.25M for three minutes. The cells were then washed with PBS, resuspended in lysis buffer (1% SDS, 20mM HEPES pH 7.6 and protease inhibitors (Merck)) to a concentration of 15 million cells/mL and sonicated using a Diagenode Bioruptor Pico sonicator (Diagenode, Denville, USA) using 7 cycles of 30 seconds on, 30 seconds off. The sonicated chromatin was snap frozen and stored at -80°C until further processing (described below).

#### RNA sequencing

We performed RNA sequencing in a subset of 10 SMuRFless MI patients and 10 controls. To extract RNA from TRIzol, we used the TRIzol/RNeasy hybrid protocol. Per 1 mL of TRIzol 200 μL chloroform was added and incubated for 5 min at room temperature after thorough mixing. After centrifugation for 15 min at 12000 g at 4°C, the upper aqueous phase was transferred to a RNAse free Eppendorf tube and mixed with an equal volume of 70% ethanol. The sample was loaded onto RNeasy mini columns (Qiagen, Hilden, Germany) and further processed according to the manufacturer’s protocol. To elute the RNA, 30 μL of RNase free water was added to the columns, incubated for 1 min and spun down at max speed.

Isolated RNA was sequenced by the Beijing Genomics Institute. Low-quality filtering and adapter trimming were performed using Trim Galore! v0.4.5 (Babraham Bioinformatics).^36^ Reads were mapped to the human reference genome (GRCh38.95, Ensembl) with Star v2.7.5a,^37^ resulting in BAM files. BAM files were counted with HTSeq [HTSeq-count tool v0.11.0]^38^ with default parameters using the complementary gtf file, containing annotation for GRCh38.95 (Ensembl). Prefiltering of the data was performed, requiring ≥10 counts in 75% of samples, followed by data normalization using DESeq2’s median of ratios.^39^ Differential expression (DE) analysis was then performed using DESeq2. The threshold of FDR adjusted p-value ≤ 0.05 was applied in declaring significant DE genes.

#### Chromatin immunoprecipitation

Chromatin immunoprecipitation (ChIP) was performed on sonicated chromatin using antibodies targeting tri-methylation of lysine 4 on histone H3 (H3K4me3) (Diagenode), H3K9me3 (Diagenode), and H3K27me3 (Diagenode). Thirty three μL of the sonicated chromatin lysates was incubated with dilution buffer (1.0% Triton-X-100, 167 mM NaCl, 1.2 mM EDTA, 16.7 mM Tris pH 8.0 in MiliQ), 12 μL protease inhibitor cocktail (Merck) and 1μg of H3K4me3, H3K9me3 or H3K27me3 antibody to a final volume of 300 μL, and incubated overnight at 4°C with rotation. Twenty μL of washed magnetic Dyna beads protein A/G (Invitrogen) was added and rotated for 60 min at 4°C. Beads were then washed one time with 500 μl washing buffer containing 0.1% SDS, 1% Triton-X-100, 150 mM NaCl, 2 mM EDTA, and 20 mM Tris pH 8.0 for 5 minutes at 4°C. Two times with wash buffer containing 0.1% SDS, 1% Triton-X-100, 500 mM NaCl, 2 mM EDTA, 20 mM Tris pH 8.0 for 5 minutes at 4°C. Lastly, beads were washed two times with wash buffer containing 1.0 mM EDTA and 10 mM Tris pH 8.0 for 5 minutes at 4°C. After washing, chromatin was eluted using 200 μl elution buffer (1% SDS, 0.1 M NaHCO3 in MiliQ) for 20 minutes at RT. Supernatant was collected and 8 μl 5M NaCl and 2 μl proteinase K (10 mg/mL) (Qiagen) were added. A separate input sample was prepared using the same ratio elution buffer, proteinase K and NaCl. ChIP and input samples were de-crosslinked for 4h at 65°C while shaking at 1000 rpm. Finally, samples were purified using the Qiaquick MinElute PCR purification kit (Qiagen) and eluted in 20 μl buffer EB (Qiagen). The ChIP-DNA was evaluated using SYBR Green (Applied Biosciences) quantitative polymerase chain reaction (qPCR) targeting the promoters of the genes coding for TNF-α, IL-6, and IL-10. Analyses of qPCR was performed using a comparative Ct method. The primers, including the positive and negative controls, used for qPCR are listed in the key resources table.

### Quantification and statistical analysis

Baseline characteristics are presented as median with interquartile range values for continuous variables, and as percentages for categorical data. Differences in baseline characteristics were tested with a Mann-Whitney U test for continuous parameters and with the chi-square test in case of categorical data. Because of the non-Gaussian distribution of most inflammatory parameters, nonparametric tests were used. Comparison between groups was performed using Mann-Whitney U test. Categorial variables are expressed as numbers (percentages) and between group differences were analyzed using chi-square test, or Fisher’s exact test, whichever appropriate. A p-value of ≤ 0.05 was considered statistically significant.

For the cytokine score, the cytokine measurements were assessed for suitability in factor analysis using Bartlett’s test of sphericity and Kaiser-Meyer-Olkin (KMO) test, resulting in the exclusion of the Pam-3-Cys IL-1β due to low sampling adequacy (KMO <0.6). Rather than creating a regression model with multiple correlated independent variables (8 cytokines), the two significant and uncorrelated principal components (as defined by Kaiser's rule with an eigenvalue greater than 1) were included. Logistic regression was performed using a binomial general linear model in R using the formular glm(y ∼ PC1 + PC2), where y is patient/control and PC1/2 are the loadings from PCA. A cytokine score for each sample was then obtained by testing the loadings of the samples against the regression model.^18^^,^^50^

For proteomics analyses, data was analyzed using unpaired Student’s t-tests and corrected using false discovery rate (FDR). A statistically significant difference was defined as a FDR corrected p-value ≤ 0.05. Heatmaps were produced by filtering the PEA data to remove NA values before plotting expression data. The cytokine score and RNA-seq analyses were performed in R,^40^^,^^51^ and the script is available on request. All other statistical analyses were performed using Prism (GraphPad software version 9.0 San Diego, USA) or SPSS (SPSS Statistics version 25, IBM Corp., Armonk, USA).

All data were visually assessed and in case of potential outliers a Grubb’s test was performed. Correction for outliers was applied in case of a p-value ≤0.05. Statistical details per analysis can be found in the figure legends.

Sex-differences were not analyzed due to the small sample size.

For some parameters, we could not include all patients, because of lack of enough material or because of technical problems. Therefore, the n for all presented parameters are provided in the figure legends.

## Data Availability

• RNA-seq data have been deposited at Gene Expression Omnibus (GEO) and are publicly available as of the date of publication. Accession numbers are listed in the key resources table. • This data does not report original code. • Any additional information required to reanalyze the data reported in this paper is available from the lead contact upon request.
