# Supplementary material for: Peripheral blood mononuclear cell hyperresponsiveness in patients with premature myocardial infarction without traditional risk factors
Source: iScience. 2023 Jun 19;26(7):107183. doi: 10.1016/j.isci.2023.107183 (PMC10338301; doi:10.1016/j.isci.2023.107183)
Supplement: Document S1. Figures S1–S5 and Tables S1–S3 [file mmc1.pdf]

iScience, Volume *26*

Supplemental information

Peripheral blood mononuclear cell hyperresponsiveness in patients with premature myocardial infarction without traditional risk factors

Jan-Quinten Mol, Julia van Tuijl, Siroon Bekkering, Charlotte D.C.C. van der Heijden, Sander A.J. Damen, Benjamin C. Cossins, Liesbeth van Emst, Tim M. Nielen, Laura Rodwell, Yang Li, Gheorghe A.M. Pop, Mihai G. Netea, Niels van Royen, Niels P. Riksen, and Saloua El Messaoudi

Supplemental information

Table S1: Patient characteristics at the time of MI. Related to Table 1.

**Patient characteristics at time of MI**	**n = 20**
Age, (years)	*47 (44-49)*
BMI, (kg/m²)	*25 (24–28)*
TChol, (mmol/L)	4.7 (4.5–5.4)
LDLc, (mmol/L)	3.3 (2.9–4.2)
HDLc, (mmol/L)	1.2 (0.9–1.4)
Triglycerides, (mmol/L)	1 (0.7–1.6)

Data are presented as median and interquartile range.

Table S2: Patient lipid levels before interruption of statins. Related to Table 1.

**Patient lipid levels before interruption of statins**	**Patients n = 20**
TChol, (mmol/L)	3,4 (3,0-3,8)
LDLc, (mmol/L)	1,8 (1,7-2,0)
HDLc, (mmol/L)	1,1 (1,0-1,3)
Triglycerides, (mmol/L)	1,2 (0,9-1,5)

Data are presented as median and interquartile range**.**

Table S3: Spearman correlations between LDLc and cytokine production after stimulation with LPS and Pam3Cys. Related to Figure 4.

	**Patients**		**Controls**	
	P-value	Spearman r	P-value	Spearman r
IL-1β LPS	0,48	0,19	0,55	0,15
IL-6 LPS	0,08	0,45	0,90	0,03
IL-10 LPS	0,35	0,25	0,69	-0,10
TNFα LPS	0,48	0,19	0,72	-0,09
IL-1Ra LPS	0,86	0,05	0,64	- 0,12
IL-1b P3C	0,11	0,41	***0,02***	0,56
IL-6 P3C	***0,02***	0,56	0,10	0,40
IL-1Ra P3C	0,20	0,34	0,25	0,29
IL-10 P3C	0,07	0,47	0,06	0,46

Figure S1: Flow cytometry uMAP marker density plots. Related to Figure 2.

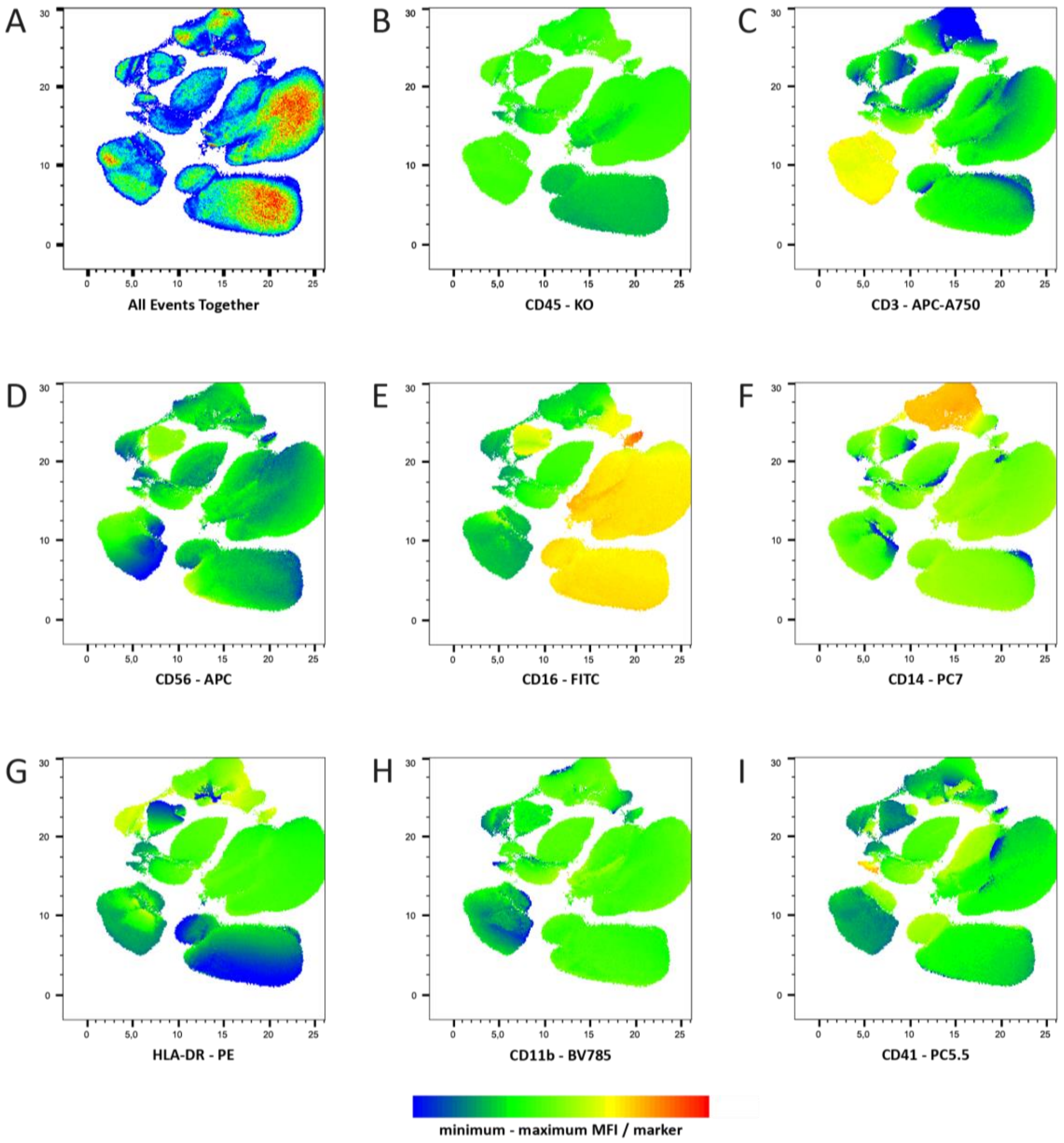

Density plots of the uMAP analysis combined for patients and controls with overlays for CD45 (B), CD3 (C), CD56 (D), CD16 (E), CD14 (F), HLA-DR (G), CD11b (H), and CD41 (I).

Figure S2: Flow cytometry manual gating strategy. Related to Figure 2.

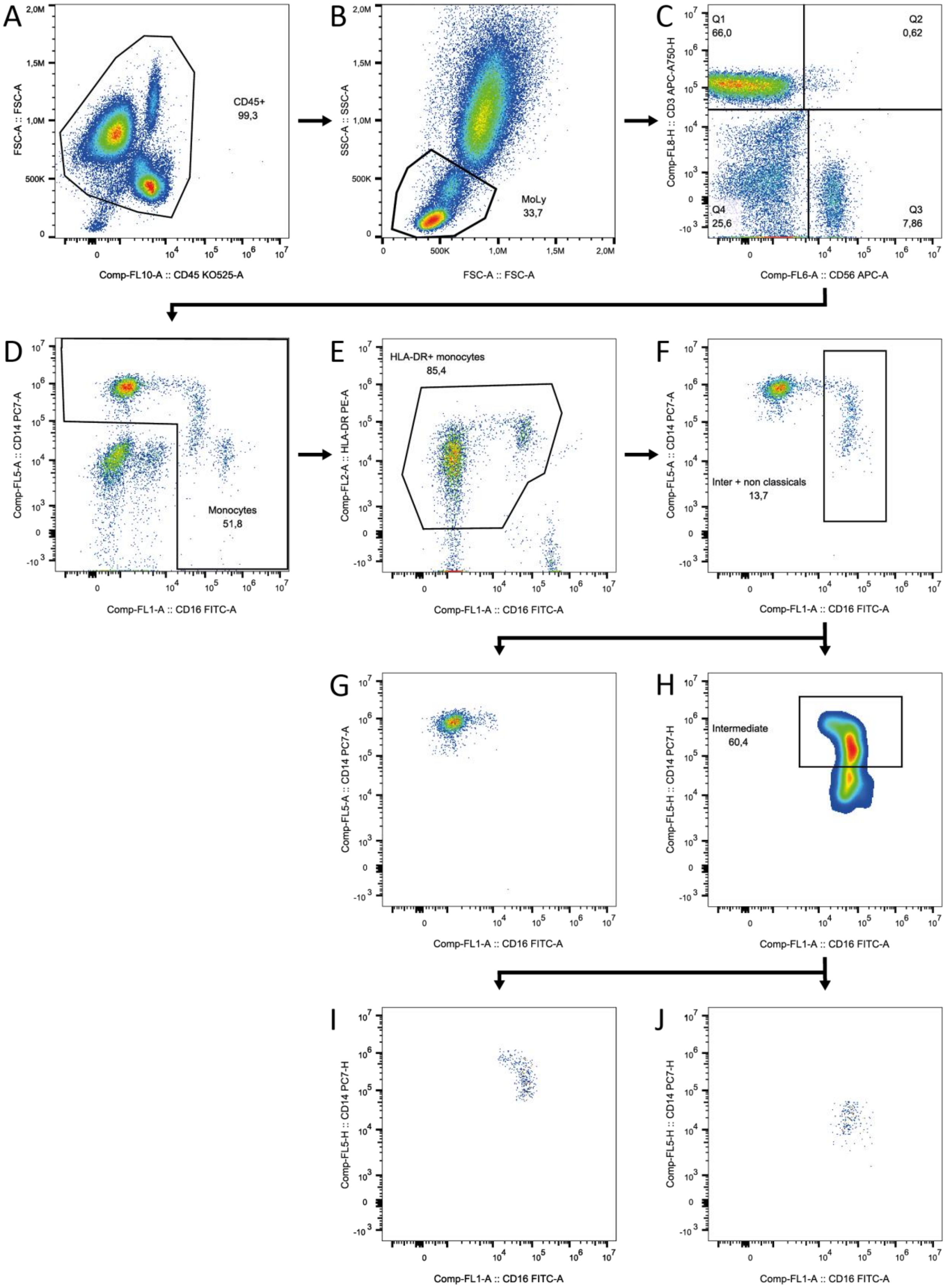

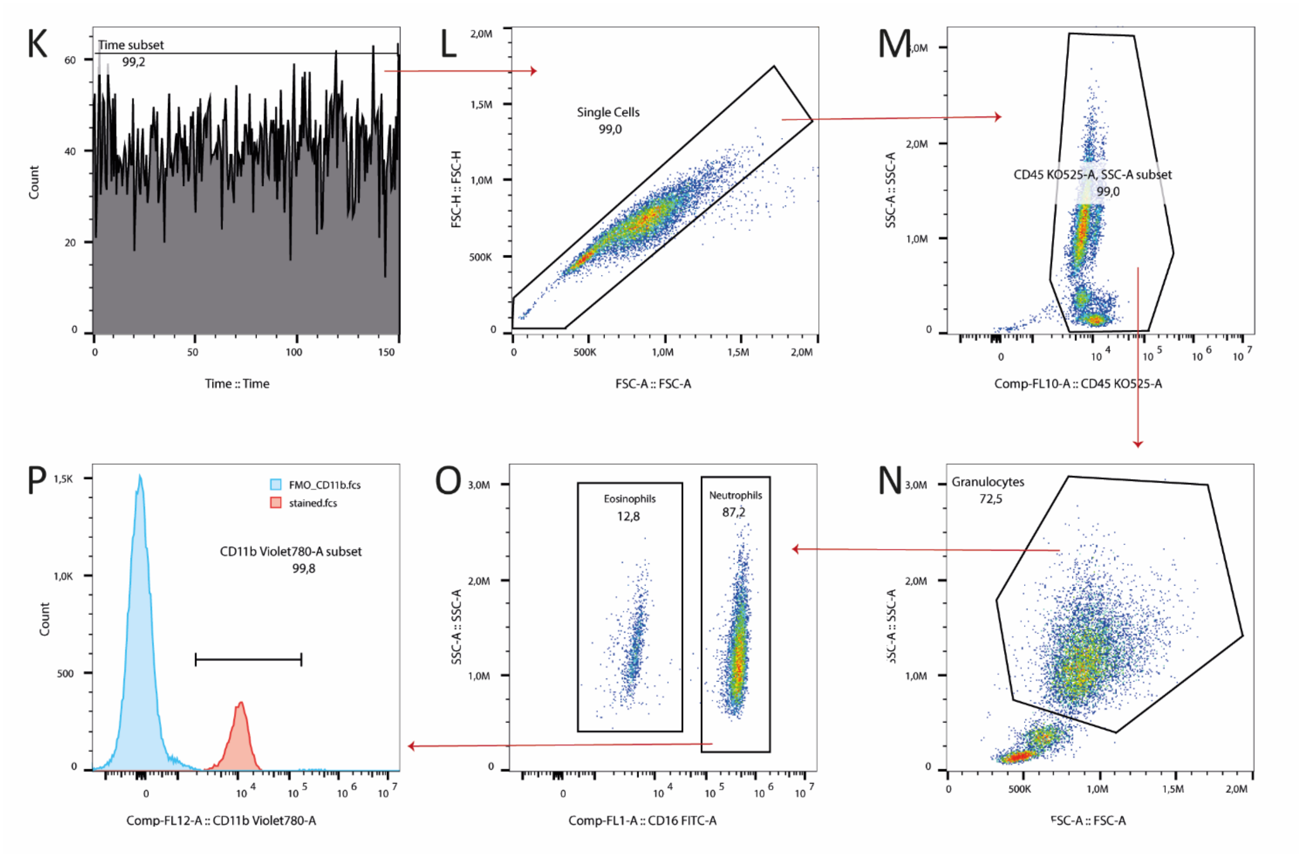

Supplemental figure 2 shows the gating strategy used in manual gating for monocytes subsets and preliminary neutrophil analysis. (A) Gating for CD45+ cells. (B) Gating for monocytes and lymphocytes based on forward- and side scatter. (C) A CD3 – CD56 plot allows for exclusion of T-lymphocytes and natural killer cells. We continued with Q4. (D) We excluded B-cells based on low expression of CD14 and CD16. (E) We excluded HLA-DR-cells. (F) In a CD14-CD16 plot we separated monocytes into a CD14++CD16- classical subset (G) and a combined CD14++CD16+ intermediate, and non-classical CD14+CD16++ monocyte subset. (H) further separation based on CD16 expression resulted in a CD14++CD16+ intermediate subset (I), and non-classical CD14+CD16++ monocyte subset (J). (K) gating strategy for neutrophil sub-analysis: gating for time, (L) singlets and (M) CD45+. Then granulocytes were selected based FSC/SSC (N). Eosinophils and Neutrophils were separated based on CD16 expression (O). Neutrophils were further analyzed for their median fluorescent intensity of CD16, CD11b and HLA-DR (P).

Figure S3: Granulocyte sub-analysis. Related to Figure 2.

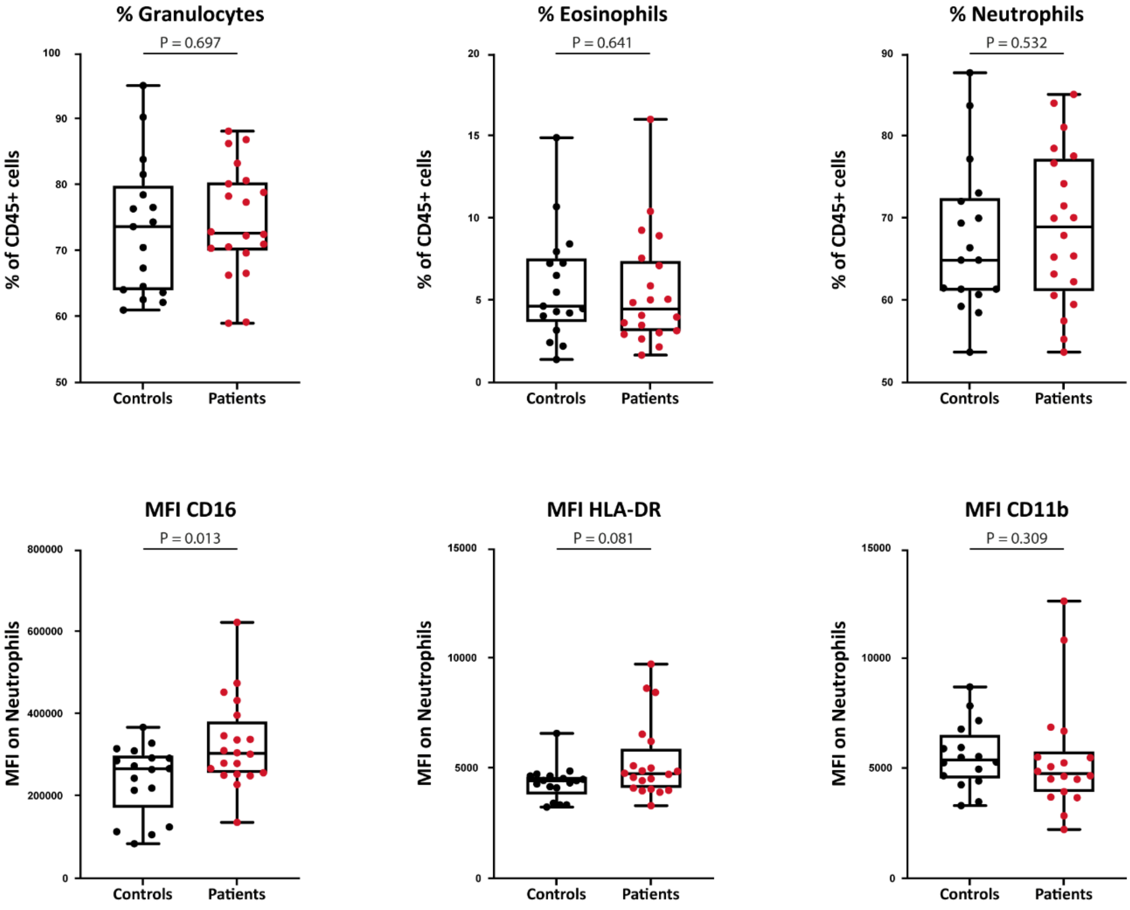

Flow cytometry granulocyte sub-analysis showed no difference in number of granulocytes, eosinophils or neutrophils between groups. CD16 expression was significantly increased on neutrophils of patients compared to controls. Data are presented as median with interquartile range. N = 17 controls vs 20 patients.

Figure S4: Individual cytokine production assays. Related to Figure 4.

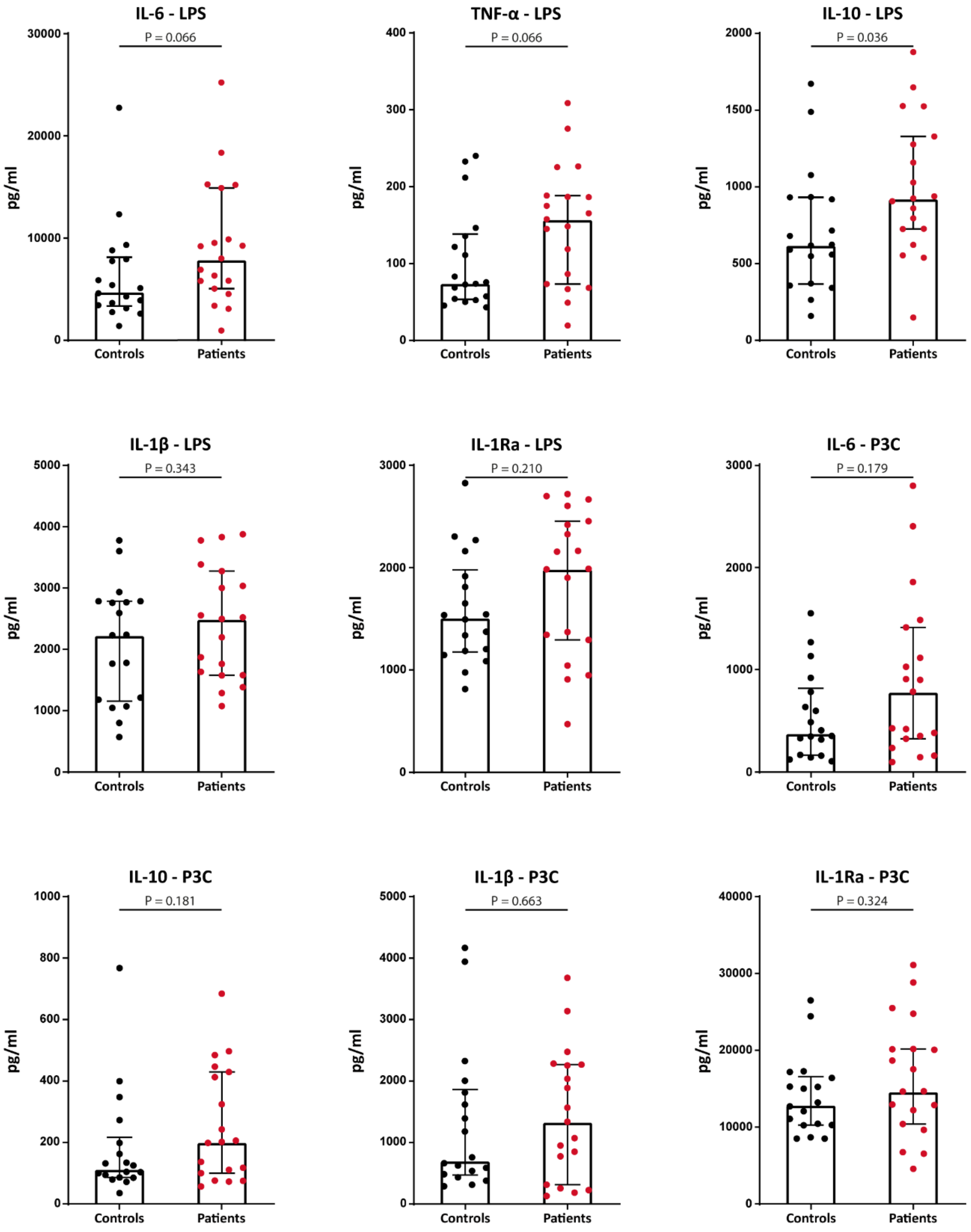

The concentrations of all individual cytokines we measured after stimulation with either LPS or Pam3Cys were consistently numerically higher in patients versus controls. Only IL-10 production after LPS stimulation reached individual statistical significance. Data are presented as median with interquartile range. N = 18 controls vs 19 patients.

Figure S5: RNA-seq differential expression analysis of inflammatory genes in unstimulated monocytes between patients and controls. Related to Figure 3.

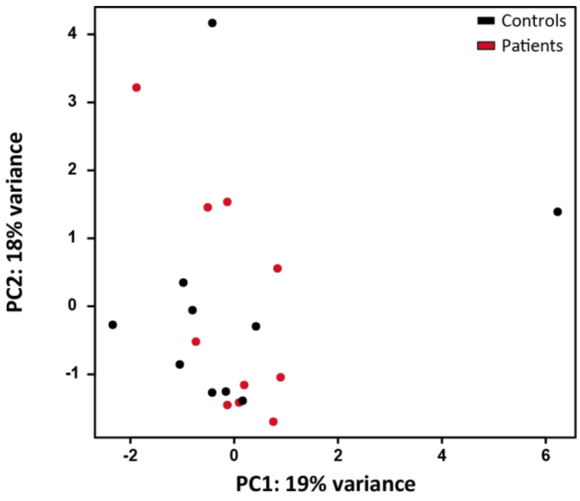

PCA plot of the 456 genes present after filtering that correspond to the inflammatory response (GO:0006954) ontology (B), did not show any differences between patients and controls. N = 10 controls vs 10 patients.
